# Supplementary material for: Excitation spillover from PSII to PSI measured in leaves at 77 K
Source: Plant Cell Physiol. 2025 Jan 7;66(3):358–73. doi: 10.1093/pcp/pcaf002 (PMC11957249; doi:10.1093/pcp/pcaf002)
Supplement: pcaf002_Supp [file pcaf002_supp.zip › suppl_data/pcp-2024-e-00244-File010.docx]

**Supplementary materials**

**Table S1 *F*II induction time, *F_m_*690, *F_m_*760, and *F_α_*760**

**Table S2 Effects of the *F*II leftover at 760 nm on fluorescence parameters**

**Fig. S1. The optical system for the fluorescence measurement.**

**Fig. S2. Transmission spectra of the 690 and 760 nm band pass filters**

**Fig. S3. The relationship between *F*690 and *F*760 induction assuming without (A) and with (B) *F*II leftover at 760 nm.**

**Text 1: Estimation of the leftover *F*II at 760 nm (with Figs. S4 and S5)**

**Text 2: Is *F*I excited by *F*II? (with Figs. S6, 7, 8 and 9)**

**Figs. S10 and S11. Electron micrographs of *A. odora* chloroplasts showing how the ratios of non-appressed thylakoid membranes to the total length of thylakoids were measured.**

**Table S1. *F*II induction time, *F_m_*690, *F_m_*760, and *F_α_*760**

+, *, **, and *** denote statistically significant differences between the data (*P* < 0.1, < 0.05, < 0.01 and < 0.001 according to *t*-test) in State 1 and those in State 2.

The effects of growth light on *t*_1/2_ are not compared statistically, because leaf materials per se were different. However, *t*_1/2_ values were consistently smaller in low light grown materials.

We confined ourselves to compare the absolute fluorescence values in the samples from the same leaves, because optical properties were different between HL and LL leaves even for the same species. Accumulation response of chloroplasts would occur in State 2 light (blue light at 10 or 5 μmol m^-2^ s^-1^). This would increase *F*II*_m_*. But, in fact, *F*II*_m_* decreased in State 2. Thus, the effect of chloroplast accumulation responses was weaker than that of the State transitions.

**Table S2. Effects of the *F*II leftover at 760 nm on the fluorescence parameters shown in Fig. 4****

*F*I*_α_* and *F*I*_β_*/*F*I*_m_* data at 0% leftover are shown in Fig. 4. When NPQ was developed, the assumption of 20% spillover resulted in a negative *F*I*_v_*/*F*I*_m_* value, indicating that the leftover was less than 20%. *F*I*_v_*/*F*I*_m_* becomes 0, when the leftover is 19.6%.

**Fig. S1. The system for *F*_v_/*F*
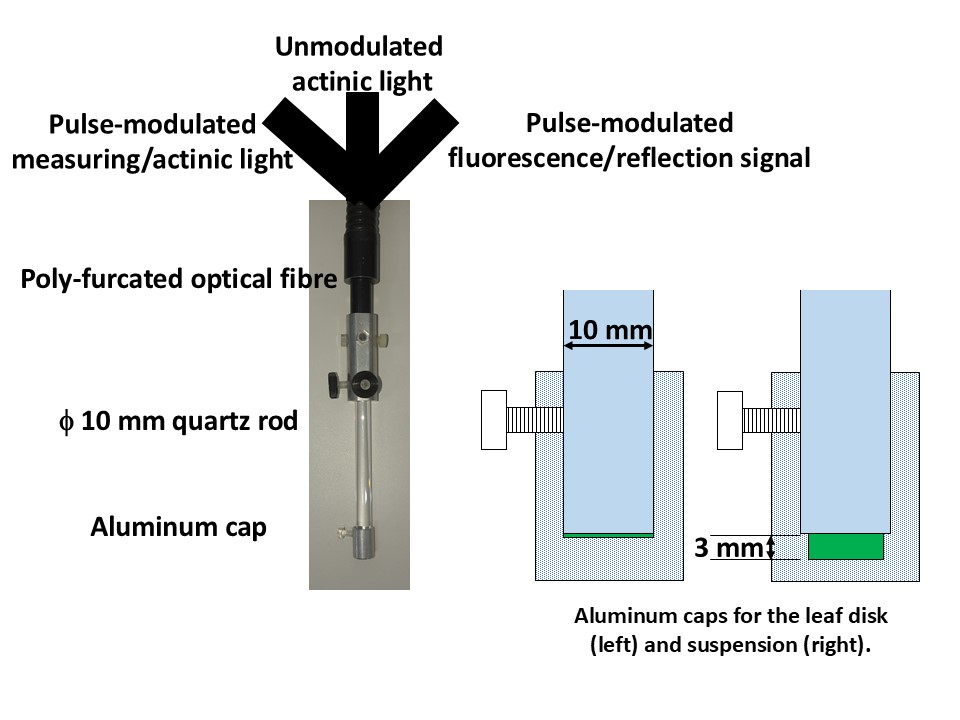
 measurement.**

**
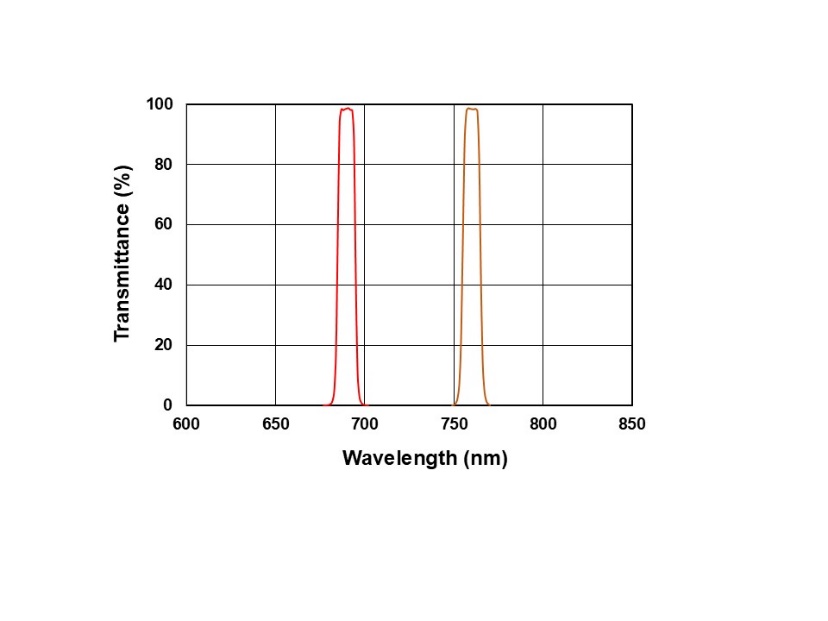
**

**Fig. S2. Transmittance spectra of the 690 and 760 nm bandpass filters.**

**The half-band width was 10 nm. The tailings were small.**

**
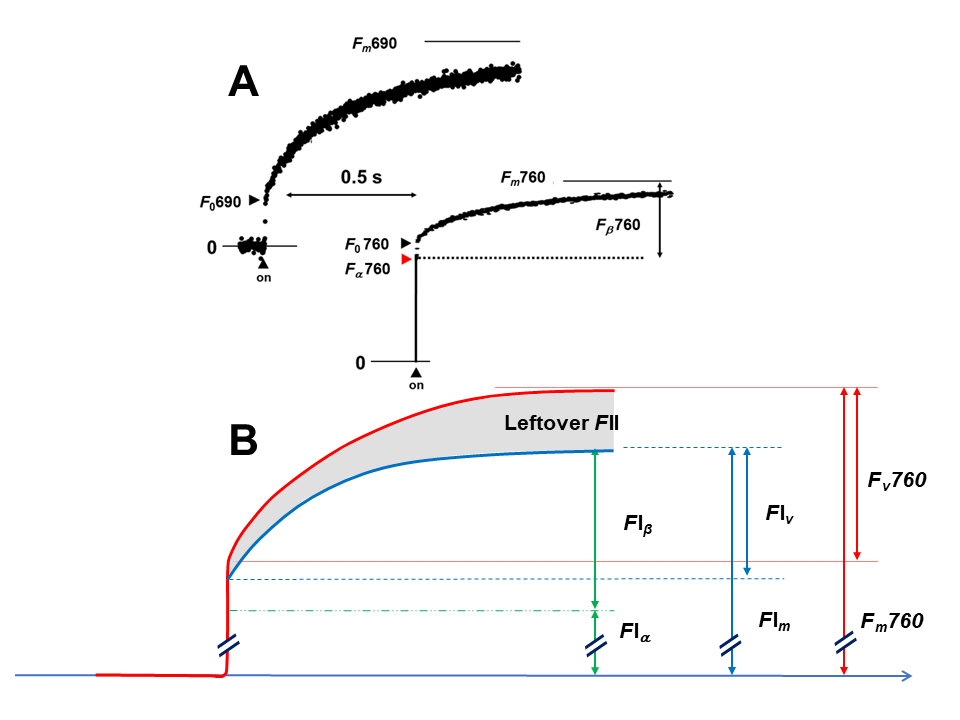
**

**Fig. S3. Interrelationships between *F*690 and *F*760 without (A) and with (B) leftover *F*II.**

When the leftover PSII fluorescence at 760 nm is absent, induction of F760 is exclusively due to the spillover from PSII. However, if there is a leftover *F*II at 760 nm, we cannot distinguish the spillover from the contamination of *F*II.

When *F*II*_m_*/*F_m_* at 760 nm is *γ*, and if we assume *RV*II = *RV*690, then equations expressing *F_m_* and *RV* can be formulated as follows:

${FI}_{m}+\gamma\cdot F_{m}=F_{m}$

$$\left( 1-\gamma\right)\cdot RVI+\gamma\cdot RV690=RV760$$

From these, *RV*I, *F*I*_α_*, *F*I*_β_* and the spillover ratio can be obtained as:

$RVI=(RV760- \gamma\cdot RV690)/\left( 1-\gamma\right)$

${FI}_{\alpha}= {(1-RV760}/{RV690})/F_{m}760$

${FI}_{\beta}= {(RV760}/{RV690}-\gamma)/F_{m}760$

${{FI}_{\beta}}/{{FI}_{m}}= {(RV760}/{RV690}-\gamma)/(1-\gamma)$.

For *F*I*_β_* >0, $\gamma$ should be less than ${RV760}/{RV690}.$ Note also that *F*I*_α_* is constant irrespective of *λ*.

**Estimation of the leftover *F*II at 760 nm**

For this series of measurements, we grew *A. odora* and *C. sativus* outdoors in the soil in the pots. The *A. odora* plant used was the clone of the plants used in other measurements. For *C. sativus,* the same variety (‘Nanshin’) was used. We purchased *S. oleracea* from a local market. The measurements were conducted in July 2024, the warmest season.

Emission spectra of *F_m_*, excited by blue light from an LED peaked at 450 nm, were measured using a photodiode array spectrophotometer (C10083CAH, Hamamatsu Photonics, Japan) and an optical system similar to that used for the *F_v_*/*F_m_* measurement as described in Terashima *et al*. (2021). *F_v_*/*F_m_* in the leaf discs were measured as described in detail in the Materials and Methods using the band pass filters. The leaf discs were treated in State 1 light at 720 nm at 10 μmol m^-2^ s^-1^ at least for 30 min. These spectra are shown in Fig. 2 in the main text.

We first assumed that the *F*II*_m_* would be expressed by a linear function for the wavelength range between 730 and 780 nm:

${F\mathrm{II}}_{m}\lambda=b-k(\lambda-730)$ Eq. S1

where $\lambda$ is wavelength in nm ($730 \leq\lambda\leq780$). Then, the *F_m_* level fluorescence at wavelength $\lambda$ can be expressed as:

${FI}_{m}\lambda+{F\mathrm{II}}_{m}\lambda={FI}_{m}\lambda+ b-k(\lambda-730)=F_{m}\lambda$ Eq. S2

We assumed that *VR*II was identical to that at 690 nm, *VR*690. Because *VR*I is unknown, *VRλ* is expressed as:

$(1-\frac{b-k\left( \lambda-730 \right)}{F_{m}})\cdot RVI+\frac{b-k\left( \lambda-730 \right)}{F_{m}\lambda}\cdot RV690=RV\lambda$ Eq. S3

We obtained the set of *b*, *k* and *RV*I that minimized the residual sum of squares (*RSS*) of the differences between the predicted and measured $F_{v}/F_{m}$values at six wavelengths:

$$RSS= \sum_{\lambda} \left[ \left( 1-\frac{b-k\left( \lambda-730 \right)}{F_{m}\lambda} \right)\cdot RVI+\frac{b-k\left( \lambda-730 \right)}{F_{m}\lambda}\cdot RV690-RV\lambda\right]^{2} Eq. S4$$

The *F*_m_ spectra shown in Fig. 2 in the main text were not corrected for the sensitivity of the photomultiplier. However, we corrected the values using the data provided by the manufacturer (Hamamatsu Photonics,
Hamamatsu Japan). Calculations were made with R statistical software (version 4.2.2; R Foundation for Statistical Computing; available from http://www.R-proje ct.org). The function *nls* was used to obtain *b*, *k*, and *RV*I by fitting a nonlinear relationship among wavelength, *F_m_* and *VR* with the least-squares method. We measured three (sometimes 2, 4 and 5) *F_v_*/*F_m_* values for each wavelength for each species. The three independent variables were estimated using all these values. For the fitting, see Fig. S4


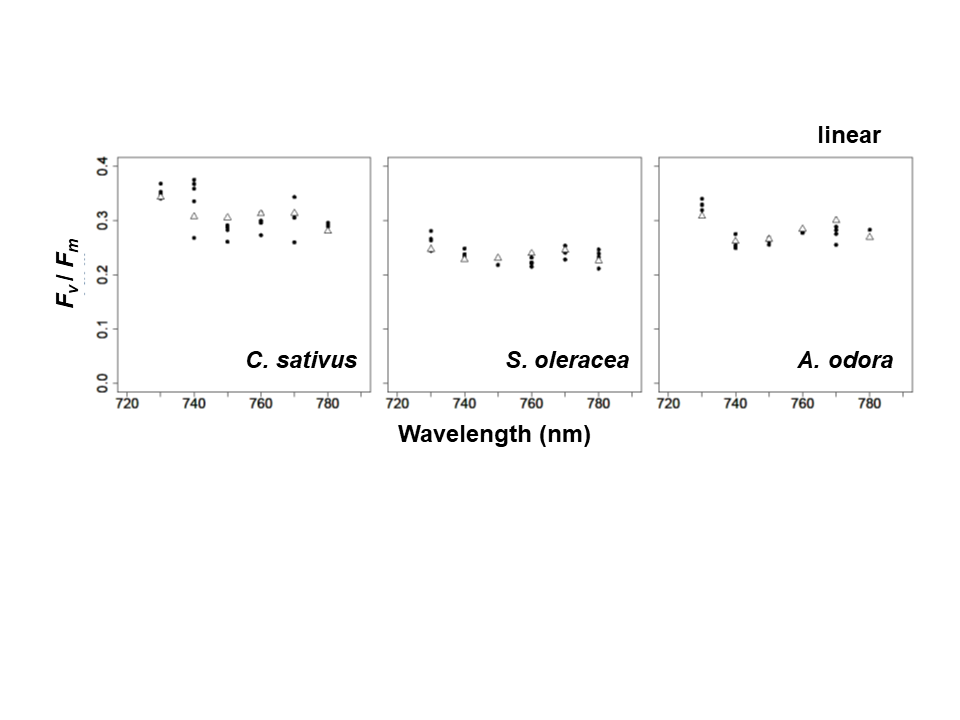


**Fig. S4. The data used for the fitting and the modelled values.**

Solid circles were the raw *F_v_*/*F*_m_ data. Open triangles were modelled *F*_v_/*F*_m_ values.

Second, to consider effects of PSII spectra bearing vibronic shoulders or peaks at around 750 nm, we digitized four such spectra of the particles (Fig. S5-A). The average of these four fluorescence spectra was well fitted by a cubic function, and as above, we obtained *RV*I and the constant for expressing *F*II*_m_λ* that minimized RSS (Table 2). However, the *RV*I value was greater than *RV*760 in all the species, and the fitting was poor (see Fig. S5-B vibronic). Among these four curves, one curve was fitted by a quadratic curve well. Using this curve, we also obtained *RV*I and a constant for *F*II*_m_λ* that minimize RSS (Table 2). At 760 nm, *F*II*_m_* was estimated to be 13% for *S. oleracea*, 14% for *C. sativus* and 18% for *A. odora*. Fitting was better than those by the linear function (Figs. S5-B). These results indicate that the compound changes in the *F_v_*/*F_m_* values would be attributed to a long tailing of *F*II*_m_* and the substantial level of *F*II*_m_* around the *F*I peak. Given that fitting by the linear functions (Fig. S4) and by the quadratic function (Fig. S5-B) was good, we may tentatively assume the leftover level of *F*II fluorescence at 760 nm would be 10 to 20%.


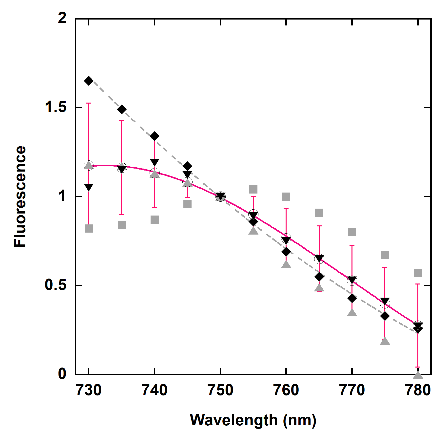


**A**

**
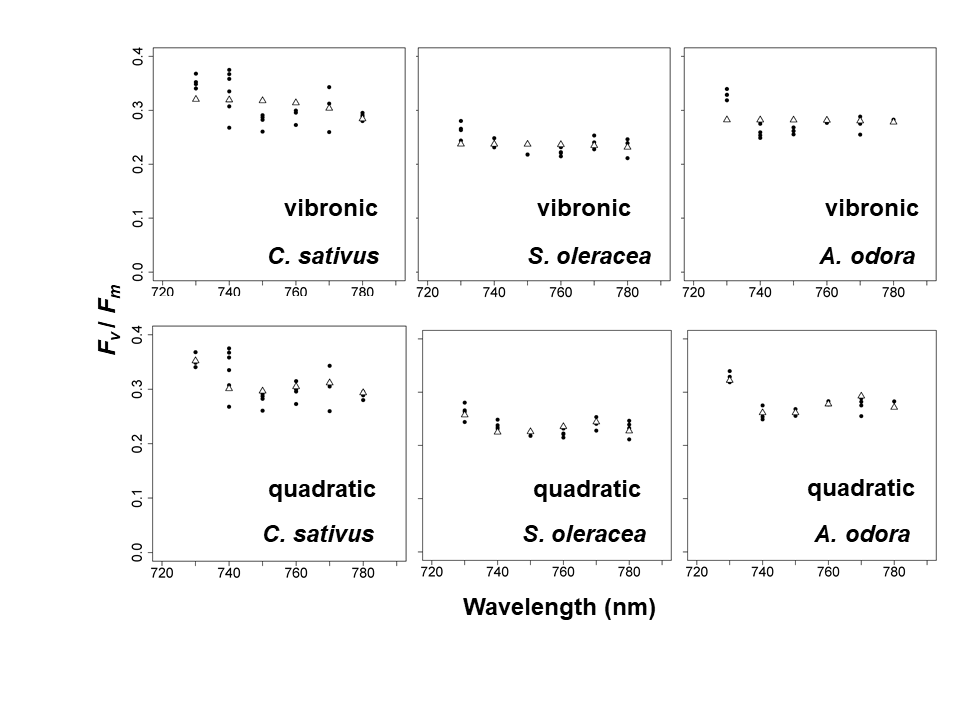
**

**B**

**Fig. S5. Digitized data of PSII particle fluorescence spectra bearing vibronic peak or shoulders (A) and fitting of the models (B).**

The averages of four curves (solid circles) were well fitted by a cubic function (red line, Y = -2822+11.062 x λ - 0.01442 x λ^2^ + 0.00000625 x λ^3^, *R*^2^ = 0.999). The curve with grey diamond symbols was well fitted by a quadratic function (interrupted grey line, Y = 120.16 - 0.28721 x λ + 0.0001711 x λ^2^, *R*^2^ = 0. 998). For , Kaleidagraph Ver. 5.04 was used.

The sources of the spectra are as follows: grey squares, *Spinacia oleracea* (van Dorssen et al. 1987); solid diamonds, *Arabidopsis thaliana* (Ruban and Johnson 2009); grey triangles, *Arabidopsis thaliana* (Karlický et al. 2016); solid inverse triangles, *Hodeum vulgare* (Karlický et al. 2016).

**Is *F*I excited by *F*II?**

Using a simple model (Fig. S5), we calculate light absorbed by a thin layer, emission of PSII fluorescence by this layer, re-absorption of this fluorescence by the rest of the leaf, and emission of PSI fluorescence caused by the absorption of PSII fluorescence, in this order. Then, the ratio of the *F*I excited by *F*II to the *F*I directly driven by the blue light will be calculated.

Transmission of the monochromatic light through a pigment solution in a cuvette is expressed by the Lambert-Beer’s law:

$-\log_{10} \frac{I_{t}}{I_{0}}=\varepsilon\cdot c\cdot l$ Eq. S5

where *I*_0_ and *I_t_* are intensities of the incident light and transmitted light, *l* is the cuvette thickness, *ε* is an absorption coefficient, and *c* is the pigment concentration. Here, we consider the cumulative pigment concentration *C* in mol m^-2^:

$C=c\cdot l$ Eq. S6

Using a simple model, let us compare PSI fluorescence directly excited by the actinic light and that excited by PS II fluorescence (see Fig. S5).


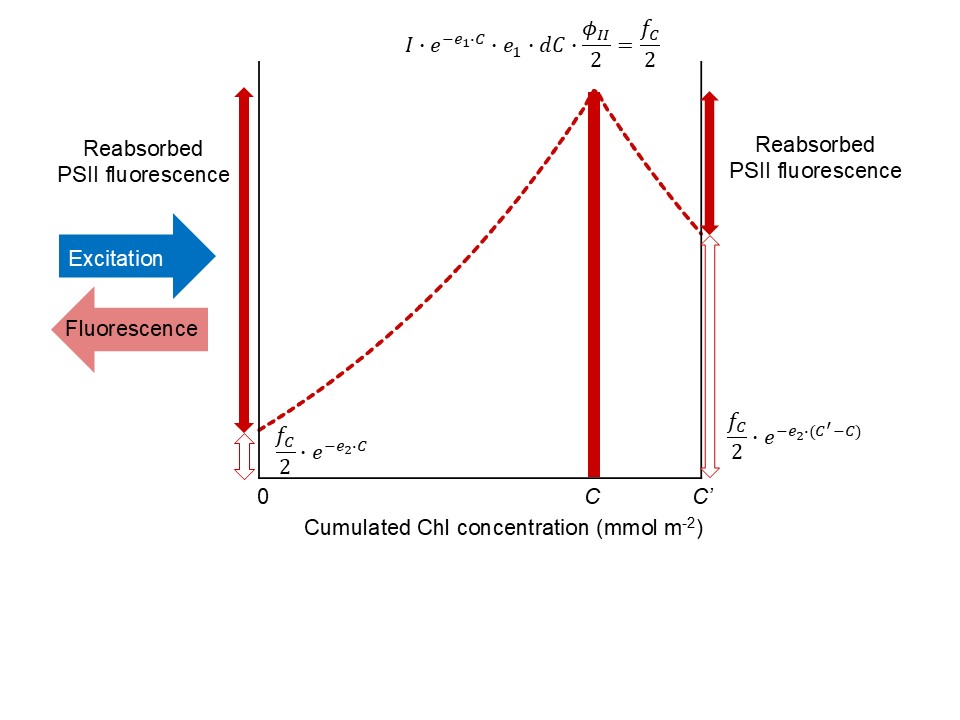


**Fig. S6. A model leaf used for estimation of PSI fluorescence excited by re-absorbed PSII fluorescence.**

A thin leaf layer *dC* absorbs light and emits fluorescence. The fluorescence emitted by *dC* is expressed as *f_C_* for simplicity.

Let us consider a thin layer (*dC*) at the cumulated chlorophyll concentration of *C* from the illuminated surface. Light absorption by this thin layer (*dA*) can be written as:

$dA=I\cdot e^{-e_{1}C}\cdot e_{1}\cdot dC$ Eq. S7

where $e_{1}$ is equal to $2.30\cdot\varepsilon_{1}$ and $\varepsilon_{1}$ is the absorption coefficient of Chl *a*+*b* at 460 nm (in m^2^ mol^-1^). Note that $\log_{e}10= 2.30$. PSII fluorescence emitted by this thin layer can be expressed as:

$dF\mathrm{II}=\phi_{\mathrm{II}}\cdot I\cdot e^{-e_{1}\cdot C}\cdot e_{1}\cdot dC$ Eq. S8

where $\phi_{II}$ denote PSII fluorescence yield.

Since PSII fluorescence emitted by a thin layer *dC* is absorbed by both sides of the layer, PSII fluorescence re-absorbed in a leaf or solution (0 < X< $C^{'})$ can be expressed as:

$dA=I\cdot e^{-e_{1}\cdot C}\cdot e_{1}\cdot dC\cdot\frac{\phi_{\mathrm{II}}}{2}\cdot[\left( 1-e^{-e_{2}\cdot C} \right)+\left( 1-e^{-e_{2}\cdot(C^{'}-C} \right)]$ Eq. S9

where $e_{2} (= {2.30\cdot\varepsilon}_{2}$) and $\varepsilon_{2} is$the absorption coefficient of Chl *a*+*b* at PSII fluorescence peak (~690 nm). PSI fluorescence excited by PSII fluorescence would not be re-absorbed. Then, PSI fluorescence from *dC* excited by PSII fluorescence and detected at the surface would be half the PSI fluorescence:

$d{FI}_{\leftarrow F\mathrm{II}}=I\cdot e^{-e_{1}\cdot C}\cdot e_{1}\cdot dC\cdot\frac{\phi_{\mathrm{II}}}{2}\cdot\frac{\phi_{I}'}{2}\cdot[\left( 1-e^{-e_{2}\cdot C} \right)+\left( 1-e^{-e_{2}\cdot(C^{'}-C} \right)]$ Eq. S10

where $\phi_{I}'$ denotes the yield of PSI fluorescence excited by PSII fluorescence. On the other hand, PSI fluorescence directly excited by the actinic light, emitted by the thin layer at *C* (*dC*), and detected by the sensor is expressed as:

$d{FI}_{direct}=\frac{\phi_{I}}{2}\cdot I\cdot e^{-e_{1}\cdot C}\cdot e_{1}\cdot dC$ Eq. S11

where $\phi_{I}$ denotes the yield of PSI fluorescence excited by the blue actinic light. Integration from 0 to $C^{'}$ of these equations give:

${FI}_{\leftarrow F\mathrm{II}}=\frac{\phi_{\mathrm{II}}}{2}\cdot\frac{\phi_{I}'}{2}\int_{0}^{C^{'}} I\cdot e^{-e_{1}\cdot C}\cdot e_{1}\cdot\left[ \left( 1-e^{-e_{2}\cdot C} \right)+\left( 1-e^{-e_{2}\cdot(C^{'}-C)} \right) \right]dC$ Eq. S12

${FI}_{direct}=\frac{\phi_{I}}{2}\int_{0}^{C^{'}} I\cdot e^{-e_{1}\cdot C}\cdot e_{1}\cdot dC=\frac{\phi_{I}}{2}\cdot I\cdot(1-e^{{-e}_{1}\cdot C^{'}})$ Eq. S13

The ratio of these integrals is expressed as:

$\frac{F_{I\leftarrow F_{\mathrm{II}}}}{F_{I direct}}=\frac{\phi_{\mathrm{II}}}{2}\cdot\frac{\phi_{I}'}{\phi_{I}}\cdot\frac{\int_{0}^{C^{'}} I\cdot e^{-e_{1}\cdot C}\cdot e_{1}\cdot\left[ \left( 1-e^{-e_{2}\cdot C} \right)+\left( 1-e^{-e_{2}\cdot(C^{'}-C)} \right) \right]dC}{\int_{0}^{C^{'}} I\cdot e^{-e_{1}\cdot C}\cdot e_{1}\cdot dC}$ Eq. S14

Because we may neglect the effects of PSII fluorescence emitted to the other side, PSII fluorescence emitted from *dC* and reaches the surface can be expressed as:

$d{F\mathrm{II}}_{surface}=\frac{\phi_{\mathrm{II}}}{2}\cdot I\cdot e^{-e_{1}\cdot C}\cdot e_{1}\cdot dC\cdot e^{-e_{2}\cdot C}$=$\frac{\phi_{\mathrm{II}}}{2}\cdot I\cdot e^{-{(e}_{1}+e_{2})\cdot C}\cdot e_{1}\cdot dC$ Eq. S15

The absorption coefficients *in situ*, *e*_1_ and *e*_2_, may be estimated by examining the effects of the cumulated Chl concentration of the thylakoid suspension on PSI and PSII fluorescence.

PSII fluorescence reaching the surface can be estimated by integrating Eq. S15:

$\frac{\phi_{\mathrm{II}}}{2}\cdot I\int_{0}^{C^{'}} e_{1}\cdot e^{-(e_{1}+e_{2} )\cdot C}\cdot dC= \frac{\phi_{\mathrm{II}}}{2}\cdot I\cdot\frac{e_{1}}{(e_{1}+e_{2} )}\cdot(1-e^{-(e_{1}+e_{2} )\cdot C^{'}})$ Eq. S16

By curve fitting of the dependence of PSI and PSII fluorescence (Eqs. S13 and S16) on $C^{'}$, *e*_1_ and *e*_2_ may be estimated (see Fig. S6 for the estimation of *e*_1_).

The ratio (Eq. S14) is a function of PSII and PSI fluorescence yields and absorption coefficients. The ratio increases with $C^{'}$ (Fig. S9). Since PSII fluorescence little excites PSII fluorescence while preferentially excites PSI, $\phi_{I}^{'}$ would be twice of $\phi_{I}$. Then, Eq. A10 may become:

$\frac{F_{I\leftarrow F_{\mathrm{II}}}}{F_{I direct}} \approx\phi_{\mathrm{II}}\cdot\frac{\int_{0}^{C^{'}} I\cdot e^{-e_{1}\cdot C}\cdot e_{1}\cdot\left[ \left( 1-e^{-e_{2}\cdot C} \right)+\left( 1-e^{-e_{2}\cdot\left( C^{'}-C \right)} \right) \right]dC}{\int_{0}^{C^{'}} I\cdot e^{-e_{1}\cdot C}\cdot e_{1}\cdot dC}$ Eq. S17

Hence, $\phi_{\mathrm{II}}$ is the key factor. The ratio of the integrals in the realistic $C^{'}$ range is at most 1~2 (Fig. S9). Because PSII fluorescence yield excited by blue light, $\phi_{\mathrm{II}}$, is small (Latimer et al. 1956, Lamb et al. 2018 and references therein), the effects of PSII fluorescence re-absorption on PSI fluorescence should be also small.

**
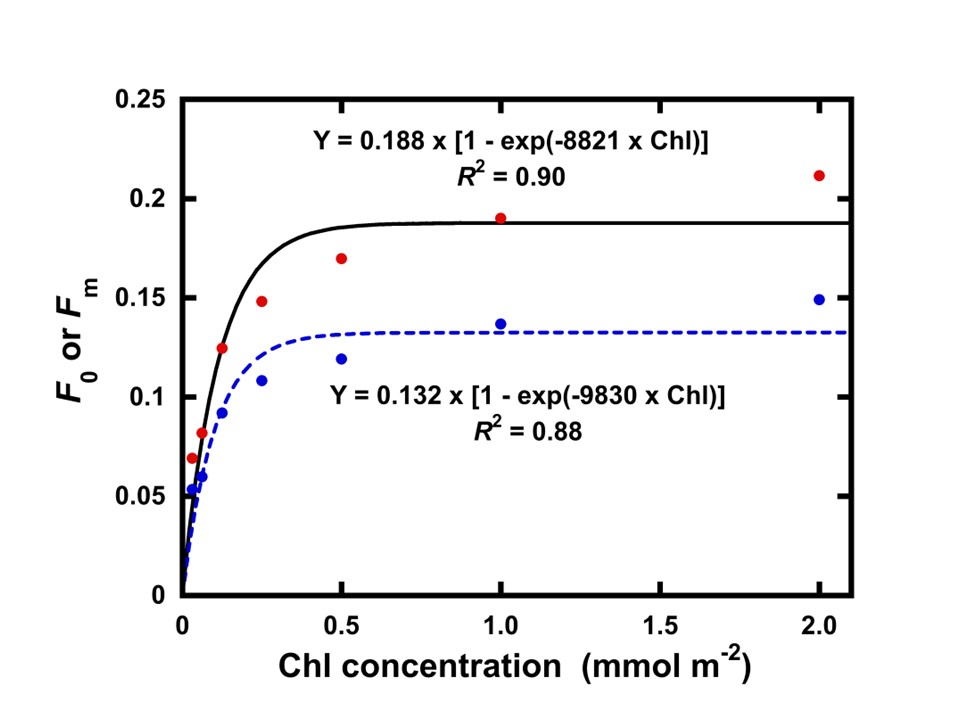
**

**Fig. S7. Determination of *e*_1_. In this calculation, we assume that no PSI fluorescence is excited by PSII fluorescence.**

**
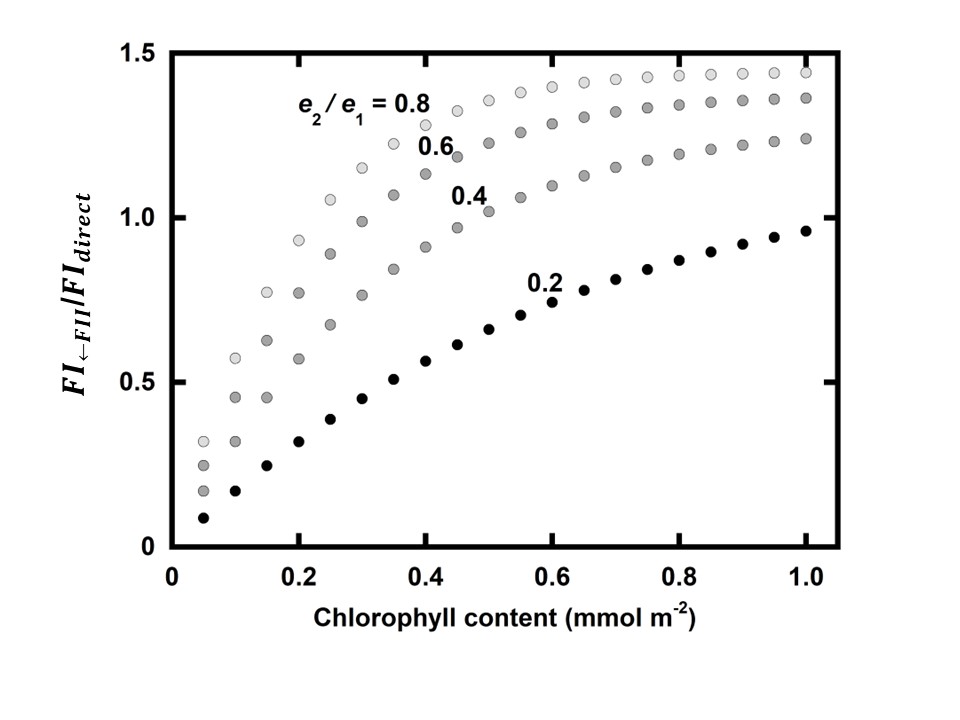
**

**Fig. S8. The ratio of the integrations in Eq. S17:**

$$\frac{\int_{\boldsymbol{0}}^{\boldsymbol{C}^{\boldsymbol{'}}} \boldsymbol{I}\boldsymbol{\cdot}\boldsymbol{e}_{\boldsymbol{1}}\boldsymbol{\cdot}\boldsymbol{e}^{\boldsymbol{-}\boldsymbol{e}_{\boldsymbol{1}}\boldsymbol{\cdot}\boldsymbol{C}}\boldsymbol{\cdot}\boldsymbol{[}\left( \boldsymbol{1}\boldsymbol{-}\boldsymbol{e}^{\boldsymbol{-}\boldsymbol{e}_{\boldsymbol{2}}\boldsymbol{\cdot}\boldsymbol{C}} \right)\boldsymbol{+}\left( \boldsymbol{1}\boldsymbol{-}\boldsymbol{e}^{\boldsymbol{-}\boldsymbol{e}_{\boldsymbol{2}}\boldsymbol{\cdot}\boldsymbol{(}\boldsymbol{C}^{\boldsymbol{'}}\boldsymbol{-}\boldsymbol{C}} \right)\boldsymbol{]}\boldsymbol{dC}}{\int_{\boldsymbol{0}}^{\boldsymbol{C}^{\boldsymbol{'}}} \boldsymbol{I}\boldsymbol{\cdot}\boldsymbol{e}_{\boldsymbol{1}}{\boldsymbol{\cdot}\boldsymbol{e}}^{\boldsymbol{-}\boldsymbol{e}_{\boldsymbol{1}}\boldsymbol{\cdot}\boldsymbol{C}}\boldsymbol{\cdot}\boldsymbol{dC}}$$

**The ratio increases with the increase in** $\boldsymbol{C}^{\boldsymbol{'}}$**. For *e*_1_, we used 9000 (namely *ε*_1_ of 9000/2.3 = 3913 m^2^ mol^-1^, see Fig. S6). We did not estimate *e*_2_ experimentally. Several possible *e*_2_ values were used (see Fig. S8).**

**
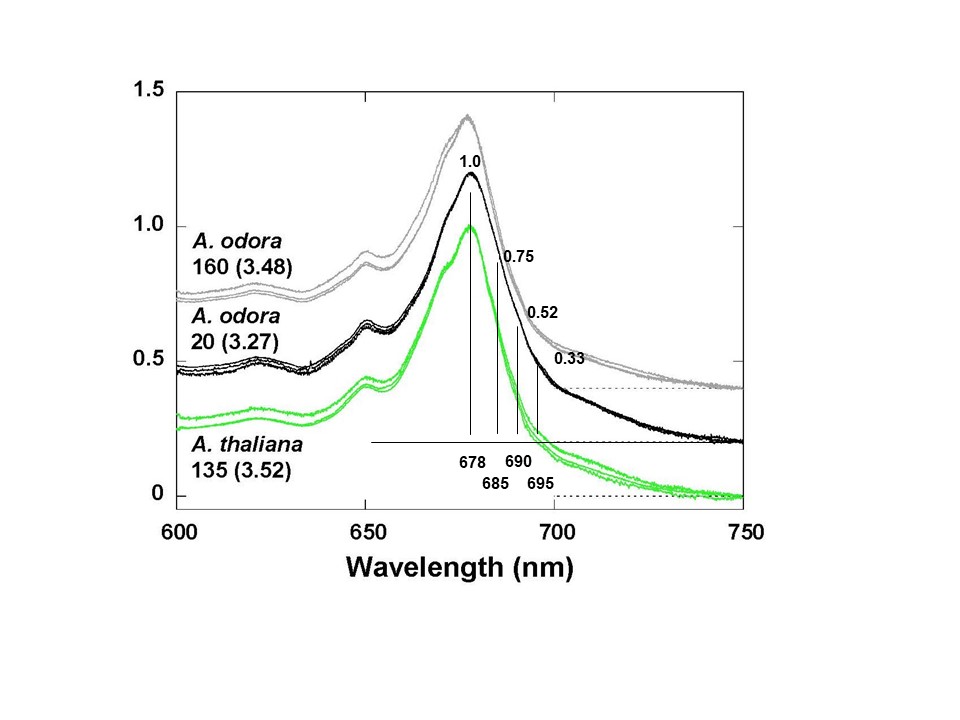
Fig. S9. 77K absorbance spectra of thylakoid suspensions prepared from the leaves of *A. odora* grown at 160 and 20, and *Arabidopsis thalian*a grown at 135 μmol m^-2^ s^-1^. Assuming the red peak (678 nm) height is similar to that at 460 nm, *e*_2_ at 685, 690 and 695 nm were estimated for low light *A. odora*. The original figure in Terashima et al. (2021) was modified.**

**
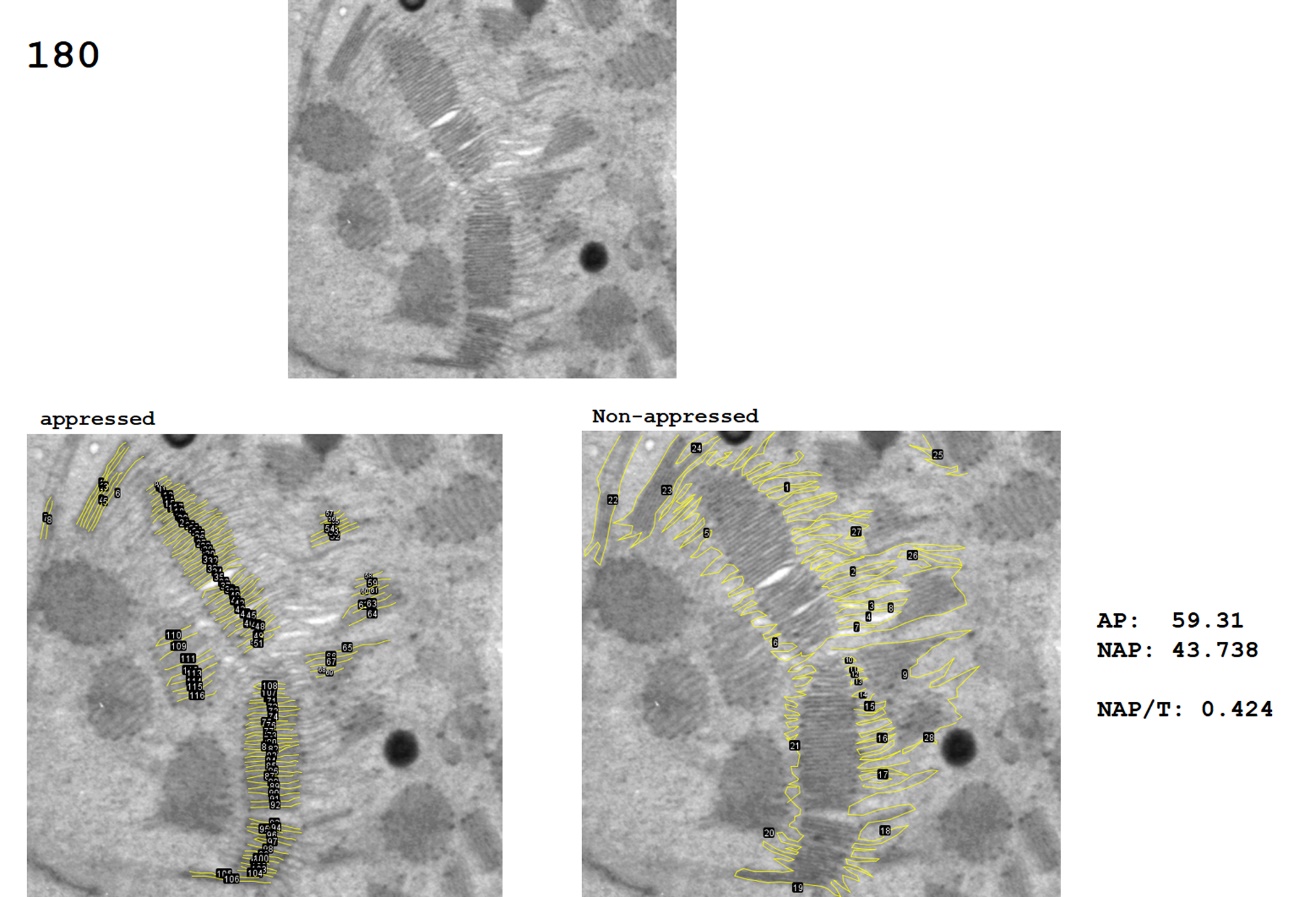
**

**Fig. S10. A part of a HL-*A. odora* chloroplast.**

Determination of the length of non-appressed thylakoid membranes (NAP) and appressed thylakoid membranes (AP). The ratio of NAP to the total length (NAP+AP) was 0.424.


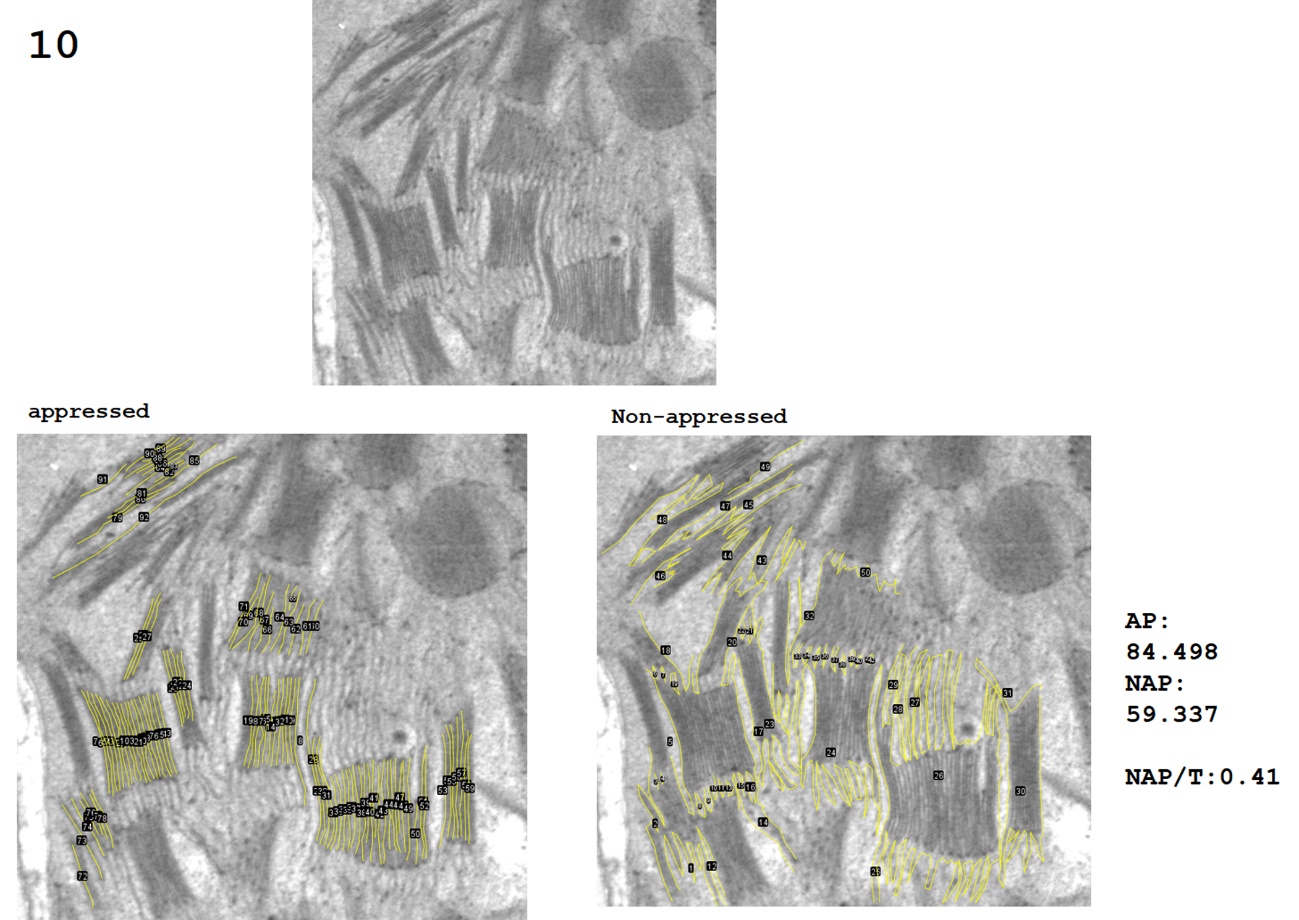


**Fig. S11. A part of a HL-*A. odora* chloroplast.**

Determination of the length of non-appressed thylakoid membranes (NAP) and appressed thylakoid membranes (AP). The ratio of NAP to the total length (NAP+AP) was 0.41.
